# Developmental coordination disorder and migraine in childhood

**DOI:** 10.1186/1129-2377-14-S1-P14

**Published:** 2013-02-21

**Authors:** M Esposito, MA Faraldo, L Antinolfi, M Carotenuto

**Affiliations:** 1 Department of Child and Adolescent Neuropsychiatry, Italy; 2 Department of Child and Adolescent Neuropsychiatry, Italy

## Purpose

Migraine without aura (MoA) could be considered the most frequent form of primary headache in children, associated with many known comorbidities, but only the recent literature has begun to consider the importance of motor impairment linked to the attacks. The developmental coordination disorder (DCD) is a very common problem among children, with a prevalence ranging up to 19%. The aim of this study was to evaluate the presence of motor coordination impairment in a population of children affected by MoA, and its role as putative risk factor for motor skills impairment.

## Methods

This observational study was performed in the Clinic of Child and Adolescent Neuropsychiatry of the Second University of Naples. MoA was diagnosed according to the International Classification of Headache Disorders (IHS-2) criteria. The study population consisted of 27 patients affected by MoA (16 females, 11 males) (mean age: 8.7 ± 2.15 years) and 59 typically developing children (34 females, 25 males) (mean age: 8.0 ± 2.1 years). The whole population underwent a clinical evaluation in order to assess the Total IQ level, the visual motor integration skills and the presence of DCD.

## Results

Our results showed that MoA children had more impairments in motor coordination (p<0.001) and visual motor integration (p<0.001) than control group**.**

## Conclusion

To our knowledge this is the first study to assess the association of poor motor coordination and MoA in children using.

## Objective measurements

These findings suggest a new perspective in the management of migraine disease in children, pinpointing that the relationship between DCD and migraine could represent a not yet understood or identified comorbidity, even if further reports are necessary, and that migraine probably could be considered not only a painful syndrome in future.
